# Quality of life of patients using intermittent urinary catheterization^1^

**DOI:** 10.1590/1518-8345.1816.2906

**Published:** 2017-07-10

**Authors:** Laís Fumincelli, Alessandra Mazzo, José Carlos Amado Martins, Fernando Manuel Dias Henriques, Leonardo Orlandin

**Affiliations:** 2 Doctoral student, Escola de Enfermagem de Ribeirão Preto, Universidade de São Paulo, PAHO/WHO Collaborating Centre for Nursing Research Development, Ribeirão Preto, SP, Brazil. Scholarship holder at Fundação de Amparo à Pesquisa do Estado de São Paulo (FAPESP), Brazil.; 3 PhD, Associate Professor, Escola de Enfermagem de Ribeirão Preto, Universidade de São Paulo, PAHO/WHO Collaborating Centre for Nursing Research Development, Ribeirão Preto, SP, Brazil.; 4 PhD, Coordinator Professor, Escola Superior de Enfermagem de Coimbra, Escola Superior de Enfermagem de Coimbra, Coimbra, Portugal.; 5 MSc, Coordinator Professor, Escola Superior de Enfermagem de Coimbra, Escola Superior de Enfermagem de Coimbra, Coimbra, Portugal.; 6 Undergraduate student in Nursing, Escola de Enfermagem de Ribeirão Preto, Universidade de São Paulo, PAHO/WHO Collaborating Centre for Nursing Research Development, Ribeirão Preto, SP, Brazil. Scholarship holder at Programa Institucional de Bolsas de Iniciação Científica (PIBIC-USP), Brazil.

**Keywords:** Urinary Bladder Neurogenic, Intermittent Urethral Catheterization, Nursing, Patient, Quality of Life, Activities of Daily Living

## Abstract

**Objectives:**

measure and compare the quality of life of neurogenic bladder patients using intermittent urinary catheterization who were going through rehabilitation in Brazil and Portugal.

**Method:**

multicenter, quantitative, cross-sectional, observational-analytic and correlational study executed in Brazil and Portugal. Two data collection tools were used, being one questionnaire with sociodemographic and clinical data and the World Health Organization Quality of Life-bref. Patients were included who were over 18 years of age, suffering from neurogenic urinary bladder and using intermittent urinary catheterization.

**Results:**

in the sample of Brazilian (n = 170) and Portuguese (n = 52) patients, respectively, most patients were single (87-51.2%; 25-48.1%), had finished primary education (47-45.3%; 31-59.6%) and were retired (70-41.2%; 21-40.4%). Spinal cord injury was the main cause of using the urinary catheter in both countries. The Brazilian patients presented higher mean quality of life scores in the psychological domain (68.9) and lower scores in the physical domain (58.9). The Portuguese patients presented higher scores in the psychological domain (68.4) and lower scores in the environment domain (59.4). The execution of intermittent urinary self-catheterization was significant for both countries.

**Conclusions:**

in the two countries, these patients’ quality of life can be determined by the improvement in the urinary symptoms, independence, self-confidence, social relationships and access to work activities.

## Introduction

The advances in health care in the global context have called for studies on the Quality of Life (QoL) of patients with chronic conditions or comorbidities that make them unable to execute the activities of daily living. In that population, the QoL outcomes reflect the patients’ ability to cope with and adapt to their new and actual living condition. In that phase, of rehabilitation, the health team’s assistance is fundamental, as care should be understood in the holistic dimension of the being, the family and the community^1^.

Studies on QoL are one way to value individual perceptions. They permit the comparison, approval and definition of treatment methods; help to understand the therapeutic choice, the assessment of costs and benefits and identify the main aspects the proposed therapeutics affect^2^. 

In the context of neurogenic bladder patients - urinary bladder dysfunction of neurological origin that alters the functioning of the bladder^3^ -, the main objectives of the treatment are the preservation of the kidney function and the person’s adaptation to the new living condition^4^. Nevertheless, the effects and implications of the treatment, such as medication use, urinary incontinence management, micturition diaries and, mainly, the use of intermittent urinary catheterization, a fundamental tool in the treatment, result in significant impacts in the activities of daily living^5^. 

Intermittent urinary catheterization is a periodical bladder voiding method, at routine intervals, by means of the introduction of a catheter through the bladder^6^. To be effective in the neurogenic bladder treatment, it should be executed regularly, which causes costs and significant changes in daily life^7^. 

In Brazil, to date, no specific policy exists for the neurogenic bladder patients who use intermittent urinary catheterization to guarantee the resources needed for high-quality care. Patients in the country are attended like all other citizens using the Unified Health System (SUS), are entitled to the material needed to execute the procedure, but this is not always available across the country^8^ ^-^ ^9^. When comparing this reality with that of other countries, changes in these policies are observed, which seem to more intensely support the use of the urinary catheter. Studies can be observed that describe the use of more advanced technologies to accomplish the procedures and strongly discuss the patient’s autonomy^10^ ^-^ ^11^. Among the different studies, due to the nature of the material found about the theme, the use of urinary catheters in Portugal stands out^12^ ^-^ ^13^.

In Portugal, a National Health System characterizes the healthcare provision. In that System, the public component guarantees care to all citizens and includes all health care, ranging from promotion to disease prevention, diagnosis, treatment and medical and social rehabilitation. In the country, the so-called “Hospital referencing network of physical and rehabilitation medicine” is in force, organized by the Directorate-General for Health (DGS), which covers the country in rehabilitation centers, located within the coverage regions. Those centers integrate multiprofesional health care and attend to the patients using intermittent urinary catheterization^14^. 

The sociocultural diversities and the health policies in force in those countries can modify the adaptation of the neurogenic bladder patients or not, who use the intermittent urinary catheterization daily, and can interfere directly in their QoL. Nevertheless, in view of this whole picture, studies are lacking that discuss the problem of intermittent urinary catheterization users. The evidence produced is focused on specific groups, such as incontinent or spinal injury patients, which does not picture the global reality^2^ ^-^ ^3^ ^,^ ^7^ ^,^ ^9^ ^,^ ^11^. 

In that sense, it is fundamental to identify the changes these patients experience with a view to effective planned actions. Among the changes deriving from the daily use of the urinary catheter, the acceptance of the new routine, changes in social life, in the work environment and treatment costs can be mentioned, among others. These patients lack specific care and effective actions, which require periodical monitoring by the health team. Particularly the nurse enables professional support in the educative, management, clinical and rehabilitation spheres and in the implementation of care plans in line with the reality the patient experiences^7^ ^,^ ^9^ ^,^ ^11^.

In that context, the objective in this study was to measure and compare the QoL of neurogenic bladder patients using intermittent urinary catheterization going through rehabilitation in Brazil and Portugal.

## Method

Multicenter, quantitative, cross-sectional, observational-analytic and correlational study executed in Brazil and Portugal. 

A convenience sample was chosen in this study. In Brazil, the data were collected at a urinary catheterization outpatient clinic of a rehabilitation center located in the interior of the State of São Paulo, between January 2014 and February 2015. At the service, patients from the city and others nearby are attended. In Portugal, the data were collected in the Central Region of the country, at two rehabilitation centers that cover this area, between September and December 2015. In the study, all patients were included who were over 18 years of age, oriented, medically diagnosed with neurogenic urinary bladder, using intermittent urinary catheterization, who practiced self-catheterization or had the procedure performed by the caregiver for more than one month. 

After the patients had formally accepted to participate by means of the Free and Informed Consent Form (FICF), the researchers collected the data by means of interviews in Brazil and Portugal. During the interview, the QoL questionnaire was self-applied or applied by the researchers by reading the questionnaire to patients with visual impairments or motor problems. 

A questionnaire was used to collect socioeconomic, clinical and procedure-related data and, to analyze the QoL, the World Health Organization Quality Life-bref (WHOQOL-bref) by the World Health Organization (WHO) was used. This tool consists of a general QoL assessment questionnaire, elaborated by the WHO Quality of Life Group (WHOQOL Group). This Likert-type scale abbreviated from the WHOQOL version contains two general questions and 24 questions representing the original questionnaire (WHOQOL-100); they are distributed in four domains: physical, psychological, social relationships and environment^15^. This tool has been translated and validated for Brazil^16^ as well as for Portugal^17^. 

The research data were coded and double-entered in Excel worksheets. The final database was imported into the application SPSS (Statistical Package for the Social Sciences), version 22 (Windows). As regards the WHOQOL-bref, the data were processed in accordance with the WHOQOL orientations to calculate the domain scores of the scale. The WHO questionnaire does not conceptually provide for the possibility to use a global QoL score. In that sense, the assessment scores were calculated in each of the four domains, as well as two general questions. The minimum score in each domain is zero and the maximum 100. The domain scores are obtained on a positive scale, that is, the higher the score, the better the QoL in that domain^16^. 

The statistical analyses developed included descriptive analyses of frequency, central trend and dispersion, inferential correlation analysis and comparison among the QoL domains and sociodemographic, clinical and urinary catheterization use characteristics of the Brazilian and Portuguese patients. Due to the non-normal distribution of the sample, for the correlations, Pearson’s correlation was used in parametric correlations and Spearman’s correlation in non-parametric correlations. To analyze the WHOQOL-bref, in the comparison with the sociodemographic, clinical and urinary catheterization usage variables, the Mann-Whitney and the Kruskal-Wallis test were used. The significance level adopted for the study was 5% (p≤0.05).

This study received approval from the Brazilian Research Ethics Committee (CONEP) (Opinion 505722/ 2013). The participants used and signed the FICF. 

The original authors of the WHOQOL-bref granted authorization for the use of the questionnaire.

## Results

In this study, 170 (100.0%) Brazilian patients and 52 (100.0%) Portuguese patients were interviewed. Among the 170 (100.0%) Brazilian patients, 121 (71.2%) were male and 49 (28.8%) female, with a mean age of 39 and a median age of 37 years. The youngest patient in Brazil was 18 years old and the oldest 95 years old.

Concerning the 52 (100.0%) Portuguese patients, 38 (73.1%) were male and 14 (26.9%) female, with a mean age of 45 years and a median age of 44 years old. The youngest patient in Portugal was 24 years old and the oldest 83 years old. The interviewees’ sociodemographic profile has been described in Table 1. 

**Table 1:** Characteristics of the participating patients according to marital status, education, family income and occupation. Ribeirão Preto, SP, Brazil, and Coimbra, Portugal, 2016

**Characteristics**		**Brazil**			**Portugal**	
		**n = 170**	**%**		**n = 52**	**%**
**Marital status**						
	**Single**	**87**	**51.2**		**25**	**48.1**
	**Married**	**56**	**32.9**		**20**	**38.5**
	**Divorced**	**17**	**10.0**		**2**	**3.8**
	**Widowed**	**6**	**3.5**		**3**	**5.8**
	**Living with fixed partner**	**4**	**2.4**		**2**	**3.8**
**Education**						
	**Illiterate**	**5**	**2.9**		**-**	**-**
	**Finished primary**	**16**	**9.4**		**-**	**-**
	**Unfinished primary**	**61**	**35.9**		**31**	**59.6**
	**Finished secondary**	**52**	**30.6**		**11**	**21.2**
	**Unfinished secondary**	**18**	**10.6**		**-**	**-**
	**Unfinished higher**	**8**	**4.7**		**-**	**-**
	**Finished higher**	**10**	**5.9**		**10**	**19.2**
**Family income**						
	<1 MW*	**5**	**2.9**		**7**	**13.5**
	1 MW*	**41**	**24.1**		**15**	**28.8**
	2-3 MW*	**79**	**46.5**		**28**	**53.8**
	3-4 MW*	**36**	**21.2**		**2**	**3.8**
	5-9 MW*	**9**	**5.3**		**-**	**-**
**Occupation**						
	**Employed**	**18**	**10.6**		**13**	**25.0**
	**Unemployed**	**12**	**7.1**		**6**	**11.5**
	**Benefit (disease aid)**	**34**	**20.0**		**8**	**15.4**
	**Retired**	**70**	**41.2**		**21**	**40.4**
	**Other** ^†^	**36**	**21.2**		**4**	**7.7**

*MW=minimum wage in Brazil. Reference value: R$788.00=US$ (209.69) (referring to Brazilian Central Bank value on 12/9/2015).

†Student, housewife, other benefit (unemployment, pensioner of the partner) and trainee.

What the origin is concerned, among the Brazilian patients, 102 (60.0%) patients came from the city where the data were collected, 55 (32.3%) from neighboring regions and 13 (8.0%) from other States. Regarding the regions of Portugal, 33 (63.5%) came from the South, 14 (26.9%) from the Central Region of the country and 5 (9.6%) from the North.

As to the Brazilian patients’ clinical characteristics, 95 (55.6%) presented spinal cord traumas, 41 (24.0%) acquired disease, 27 (15.8%) congenital disease and 7 (4.1%) iatrogenic disease. Among the Portuguese patients, 41 (78.8%) presented spinal cord trauma, 6 (11.6%) acquired disease, 3 (5.8%) congenital disease and 2 (3.8%) iatrogenic disease. 

The patients’ practice of intermittent urinary catheterization in both countries has been described in Table 2.

**Table 2:** Description of intermittent urinary catheterization used by interviewed patients. Ribeirão Preto, SP, Brazil, and Coimbra, Portugal, 2016

**Intermittent urinary catheterization**		**Brazil**			**Portugal**	
		**n = 170**	**%**		**n = 52**	**%**
**Start period of urinary catheterization**						
	**1980 till 1989**	**5**	**2.9**		**1**	**1,9**
	**1990 till 2000**	**23**	**13.5**		**7**	**13,5**
	**2001 till 2011**	**99**	**58.2**		**26**	**50,0**
	**2012 till 2015**	**43**	**25.3**		**18**	**34,6**
**Urinary catheterization frequency**						
	**2 times/day**	**8**	**4.7**		**2**	**3,8**
	**3 times/day**	**17**	**10.0**		**1**	**1,9**
	**4 times/day**	**78**	**45.9**		**15**	**28,8**
	**5 times/day**	**33**	**19.4**		**11**	**21,2**
	**6 times/day**	**25**	**14.7**		**16**	**30,8**
	**7 times/day**	**4**	**2.4**		**7**	**13,5**
	**8 times/day**	**5**	**2.9**		**-**	**-**
**Urinary catheter type**						
	**Simple**	**157**	**92.4**		**-**	**-**
	Glass*	**12**	**7.1**		**-**	**-**
	**Lubricated catheter**	**1**	**0.6**		**50**	**96,2**
	**Silicon**	**-**	**-**		**2**	**3,8**
**Urinary self-catheterization**		**107**	**62.9**		**48**	**92.3**
**Sensitivity**		**78**	**45.9**		**37**	**71.2**
**Urine loss**		**99**	**58.2**		**19**	**36.5**

*Catheter used in Brazil only.

As for the urinary catheterization inputs used, in Brazil, 152 (89.4%) patients received the material from public entities and 18 (10.6%) bought it with their own income. In Portugal, 32 (61.5%) bought the material with their own income and 20 (38.5%) received it from public entities as they were attended at the rehabilitation institutions. 

The assessment of the Brazilian and Portuguese patients’ QoL, executed by means of the WHOQOL-bref, has been described in Table 3. 

**Table 3:** Description of quality of life (WHOQOL-bref) in relation to the four domains and general questions of Brazilian and Portuguese patients according to the means, standard deviations, minima and maxima. Ribeirão Preto, SP, Brazil, and Coimbra, Portugal, 2016

**WHOQOL-bref domains**	**Brazil**					**Portugal**			
	**Mean**	**Standard deviation**	**Minimum**	**Maximum**		**Mean**	**Standard deviation**	**Minimum**	**Maximum**
**Physical**	**58.9**	**18.8**	**17.9**	**92.9**		**60.3**	**16.5**	**17.9**	**100.0**
**Psychological**	**68.9**	**18.4**	**12.5**	**100.0**		**68.4**	**16.5**	**12.5**	**100.0**
**Social**	**65.8**	**21.4**	**0.0**	**100.0**		**63.4**	**19.7**	**25.0**	**100.0**
**Environment**	**61.4**	**16.4**	**6.3**	**100.0**		**59.4**	**13.7**	**28.1**	**90.6**
QoL* (question 1)	**18.1**	**5.5**	**0.0**	**25.0**		**16.7**	**4.1**	**6.3**	**25.0**
**Health (question 2)**	**16.9**	**6.4**	**0.0**	**25.0**		**14.2**	**5.2**	**0.0**	**18.8**

*QoL - Quality of Life

The comparison of the QoL data between Brazilian and Portuguese patients indicated a statistically significant difference in the two general questions (QoL and health), as demonstrated in Table 4.

**Table 4:** Distribution of quality of life scores in relation to the domains and two general questions of WHOQOL-bref (QoL* and health) by country. Ribeirão Preto, SP, Brazil, and Coimbra, Portugal, 2016

**Country**	**Mean ranks**					
	**Physical Domain**	**Psychological Domain**	**Social Domain**	**Environmental Domain**	**Quality of life**	**Health**
**Portugal**	**115.0**	**108.4**	**103.7**	**103.8**	**94.8**	**88.3**
**Brazil**	**110.4**	**112.5**	**113.9**	**113.9**	**116.6**	**118.6**
**p** ^**†**^	**0.651**	**0.688**	**0.311**	**0.320**	**0.020**	**0.002**

*QoL - Quality of Life; †Mann-Whitney test.

The physical, psychological, social and environmental domains and the two general questions of the WHOQOL-bref (quality of life and health) were also correlated with the sociodemographic, clinical and intermittent urinary catheterization use variables by means of the Kruskal-Wallis and Mann-Whitney tests.

In Brazil, statistically significant values were found for the Brazilian patients when the marital status was correlated with the physical and psychological domain, between the occupation and the psychological and environmental domains and between who performs the procedure (patient or caregiver) and the psychological domain. In Portugal, correlations were found between who performs the procedure (patient or caregiver) and the psychological domain and the general QoL question. The significant comparisons of the Brazilian and Portuguese patients have been described in Table 5.

**Table 5:** Distribution of quality of life scores (WHOQOL-bref) of Brazilian and Portuguese patients related to marital status and occupation. Ribeirão Preto, SP, Brazil, and Coimbra, Portugal, 2016

**Variables**		**Physical Domain**	**Psychological Domain**	**Social Domain**	**Environmental Domain**	**Quality of life**	**Health**
		**Mean Ranks**					
		**Brazil**					
**Marital status**							
	**Single**	**99.2**	**94.7**	**88.2**	**90.5**	**89.7**	**93.3**
	**Married**	**66.7**	**70.3**	**83.8**	**73.9**	**79.1**	**72.9**
	**Divorced**	**81.6**	**83.0**	**75.2**	**96.4**	**81.0**	**91.5**
	**Widowed**	**51.4**	**55.0**	**67.9**	**66.6**	**74.2**	**65.4**
	**Fixed partner**	**118.6**	**154.7**	**120.7**	**121.5**	**118.5**	**96.5**
	**x** ^**2**^	**19.7**	**18.7**	**3.9**	**7.9**	**4.5**	**8.1**
	p*	**0.001**	**0.001**	**0.412**	**0.095**	**0.343**	**0.089**
**Occupation**							
	**Employed**	**110.3**	**121.1**	**112.1**	**118.2**	**98.4**	**113.3**
	**Unemployed**	**108.3**	**97.9**	**97.9**	**97.9**	**91.2**	**96.4**
	**Benefit**	**79.4**	**76.7**	**79.6**	**71.9**	**83.5**	**84.7**
	**Retired**	**79.9**	**81.0**	**80.1**	**83.1**	**89.2**	**79.3**
	**Other**	**82.2**	**80.6**	**84.1**	**82.1**	**71.8**	**80.8**
	**x** ^**2**^	**8.7**	**12.3**	**7.5**	**11.7**	**5.4**	**8.6**
	p*	**0.067**	**0.015**	**0.111**	**0.020**	**0.247**	**0.071**
**Procedure**							
	**Self-catheterization**	**89.9**	**90.8**	**87.6**	**85.2**	**86.6**	**87.4**
	**Caregiver**	**76.6**	**74.9**	**80.5**	**84.3**	**82.3**	**80.8**
	**p** ^†^	**0.089**	**0.042**	**0.355**	**0.936**	**0.552**	**0.369**
		**Portugal**					
**Procedure**							
	**Self-catheterization**	**27.9**	**27.2**	**27.0**	**27.3**	**28.0**	**26.9**
	**Caregiver**	**10.0**	**18.4**	**20.3**	**17.3**	**8.5**	**21.8**
	**p** ^†^	**0.023**	**0.261**	**0.385**	**0.202**	**0.006**	**0.479**

* Kruskal-Wallis test; † Mann-Whitney test.

## Discussion

According to WHO, QoL is “individuals’ perceptions of their position in life in the context of the culture and value systems in which they live and in relation to their goals, expectations, standards and concerns”^15^. The QoL domains adopted by WHO (physical, psychological, social and environmental) affect the general sphere of life, that is, satisfactions, aspirations, experiences, social relationships and wellbeing of an individual and the community (s)he is inserted in^18^. In that context, the alterations in the trajectory of the activities of daily living for neurogenic bladder patients using intermittent urinary catheterization demonstrate significant changes in all domains of their lives. 

In this study, the sociodemographic and clinical profiles found in the sample of Brazilian and Portuguese patients (Table 1) support results from earlier studies^19^ ^-^ ^20^. In the two samples, spinal cord injury was the main cause of using the urinary catheter. This injury is a permanent or temporary condition that affects the motor, sensitive and autonomic functions with significant biopsychosocial changes, which can influence the basic human needs, degrees of dependence and, consequently, the patient’s QoL at home^19^. A large number of patients are affected by traumatic and non-traumatic spinal cord injuries around the world. In Brazil, about 40 new cases per million inhabitants happen each year^21^. In Portugal, no recent incidence and prevalence data have been published^22^, although important recent demographic changes are known that indicate that the age at the time of the injury has strongly increased, leading to a growing number of people with spinal cord injuries. 

Like in relation to the other morbidities found, in case of spinal cord injury, intermittent urinary catheterization is the main treatment form of the changes the occurrence of the neurogenic bladder brings about in the urinary system. After the start of the procedure, it is undertaken at four to six or six to eight-hour intervals, according to the diuresis volume presented in micturition diary records and after assessment by means of urodynamic, radiologic and ultrasound tests^3^. Those results were also found in most of the patients in this sample (Table 2).

The procedure provides for bladder reeducation and favors stimuli towards safe and effective spontaneous micturition^5^. Nevertheless, it causes some inconveniences, such as the sensitivity in the introduction of the urinary catheter and urine loss in between the urinary catheterizations, as reported in both populations (Table 2). In that sense, the professionals’ support is extremely important: clear and objective therapeutic communication is needed, as well as continuing assessment of the patients’ conditions with a view to the success of the therapeutics at home^7^. 

For the success or failure of the technique and the patient’s compliance, the quality of the material used to execute the procedure is important. In this respect, differences were observed in the sample, in the acquisition process as well as in the quality of the material. The use of the lubricated catheter is not a Brazilian reality yet^9^. Nevertheless, the studies demonstrate that this type of material minimizes the user’s sensitivity and reduces the attrition with the urethral mucosa, leading to less pain and greater treatment compliance and improving the patient’s QoL by about 30.0%^4^ ^,^ ^7^. 

Concerning the QoL of the patients in this study, as demonstrated in Table 3, in the data obtained from Brazilian patients, higher QoL scores were observed in the psychological domain and lower scores in the physical domain. In the Portuguese patients, higher scores were verified in the psychological domain and lower scores in the environmental domain. In view of these data, in comparison with the standardized data for Portugal, agreement was observed for the results in the environmental domain (lower score) and disagreement in the higher scores, as the standardized data present higher scores in the physical domain for the Portuguese population^23^. What the Brazilian standardized data are concerned, in a study undertaken in the South, agreement was demonstrated with the results found in the physical domain (lower score) and disagreement in the psychological domain. In the study, the highest score was found in the social domain^24^. 

As regards the study developed in the South of Brazil, the data obtained from participants with chronic conditions presented lower scores in all QoL domains when compared to the general population^24^. Concerning the study of urinary catheterization users’ QoL, other researchers have found data similar to this study in the lower scores measured by the WHOQOL-bref^15^, as well as in the higher scores measured in the psychological domain^1^ ^,^ ^25^.

It is clear that, the greater the health team’s support for the multidimensional aspects of life in patients using clean intermittent catheterization, the better the treatment compliance will be^5^. In that process, the nurse is a decisive professional for the patient’s progress, as she helps the patient to gain independence, within the reality experienced through self-care, from the help to obtain a better QoL^1^. 

In this study, the analysis of the relation between the independent variables and each QoL domain and the two general QoL questions in the WHOQOL-bref^15^ demonstrate, in Brazil, a significant relation between the marital status and the physical and psychological domains, as patients who were single or lived with a fixed partner presented a better QoL. The relationships are one of the aspects the multiprofessional team needs to address, as the intermittent urinary catheter user can present constraints, stress, anxiety, repression during a relationship, issues that can turn into barriers for the evolution of the treatment and limitations for a better QoL^1^ ^,^ ^4^.

In Brazil, another relevant finding was the significantly positive statistics for the occupation and the psychological and environmental domains according to Table 5. The Brazilian employed patients presented a higher QoL, demonstrating the importance of these individuals’ participation in work activities and of their accessibility in public spaces. Political and health access issues tend to affect the accessibility, as exemplified by the lack of public bathrooms (inappropriate facilities), and by social issues such as feelings of pain, shock, fear, depression and difficulties to adapt to the treatment^7^. 

The collected data demonstrated the noteworthy presence of the caregiver in Brazil (37.1%), differently from the Portuguese reality (7.7%). In that country, it could be observed that the patients who practiced intermittent urinary self-catheterization presented higher QoL scores in the psychological domain. What the data collected in the Portuguese sample is concerned, that information was statistically relevant, demonstrating that the users of intermittent urinary self-catheterization presented higher QoL scores in the physical domain and in the question about QoL.

According to the WHOQOL Group, the psychological domain involves the aspects of satisfaction, wellbeing, self-esteem and negative feelings. The physical domain covers aspects related to pain, discomfort, ability to work and dependence. The general question about QoL classifies the concept as very bad, bad, neither bad nor good, good and very good^15^. Therefore, these results confirm the impact in the psychological and physical domains and QoL classification for the self-realization of the procedure, as it outlines the feelings of independence, self-sufficiency and better self-esteem, expressed by the patients.

Concerning the QoL in both countries, the two general QoL and health questions were statistically significant, as the Brazilian patients presented higher QoL scores. Those questions examine how a person assesses his general QoL, health and wellbeing^15^. In that sense, the importance of the health professionals’ work is clear, especially of the nurse, who plays an essential role in the execution, orientation, demonstration, supervision and reassessment of these patients’ conditions for self-care or care by the caregivers. 

Being a global instrument, the WHOQOL-bref made it possible to measure, in two countries with linguistic similarities but distinct environmental and cultural characteristics, the QoL. Nevertheless, more specific tools need to be developed for that population, which could complement the results obtained and can be considered as a limiting factor in this study.

## Conclusion

In the research findings in both countries, it could be identified that the improvement in the urinary symptoms, as well as the independence, self-confidence, social relationships, access to work activities and social insertion can determine the QoL of neurogenic bladder patients using intermittent urinary catheterization. As the results found are limited to regional populations, this research needs to be expanded to obtain the global characteristics of the target population. 

In that context, the health professionals need to understand this phenomenon, aiming for these patients’ satisfaction with life and with the efficacy of the support and treatment processes for neurogenic bladder patients using intermittent urinary catheterization. 
